# Season over plant sex: drivers of leaf damage and plant defence in a dioecious Mediterranean shrub

**DOI:** 10.1111/plb.70115

**Published:** 2025-09-15

**Authors:** E. Valdés‐Correcher, G. Calvo, C. Rigueiro, B. Lago‐Núñez, P. Jordano, X. Moreira

**Affiliations:** ^1^ Department of Ecology and Evolution Estación Biológica de Doñana, EBD‐CSIC Sevilla Spain; ^2^ Misión Biológica de Galicia (MBG‐CSIC) Pontevedra Galicia Spain

**Keywords:** dioecious, Doñana National Park, insect herbivory, phenolic compounds, *Pistacia lentiscus*

## Abstract

- In dioecious plants, females often prioritize reproduction over growth, potentially investing more in defence, while males grow faster but allocate fewer resources to defence, making them more susceptible to herbivory. Recent studies challenge this view, showing that males may grow more slowly and sometimes invest equally or more in defence. Variability in sex‐specific herbivory and defence strategies may stem from seasonal shifts in resource allocation, with females prioritizing growth early in the season and reproduction later. These changes complicate herbivory patterns, necessitating research that considers temporally dynamic factors.
- This study investigated plant sex influence on herbivory and defence mechanisms in *Pistacia lentiscus* over the course of a year in Doñana National Park. We assessed insect herbivory and leaf traits linked to herbivore resistance, including phenolic compounds and specific leaf area (SLA), in 100 *P. lentiscus* plants (53 female, 47 male) at two sites during early and late seasons.
- Herbivory was higher in males than females and increased late in the season. A significant interaction between plant sex and season revealed that males experienced more herbivory late in the season, while there was no significant difference in the early season. Leaf phenolic concentration and SLA were higher early in the season, but these traits were not influenced by plant sex or the interaction between plant sex and season. Moreover, plant sex and season effects on herbivory remained significant even after controlling for leaf phenolics and SLA as covariates, indicating that these traits do not fully explain the observed differences in herbivory across sexes and seasons.
- Overall, our findings highlight the complex interplay between seasonality and plant sex in shaping herbivory and defence strategies, emphasizing the need to consider temporal dynamics when studying plant–herbivore interactions in dioecious species.

## Graphical abstract

Considering both seasons revealed higher late‐season herbivory in males than in females of *Pistacia lentiscus*, while overall herbivory increased and traits varied seasonally.

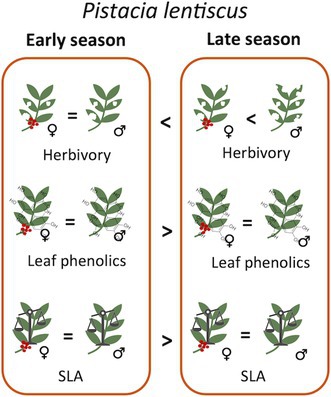

## INTRODUCTION

Sexual dimorphism in dioecious plants has a key ecological role by promoting genetic diversity within populations (Ashman 2002; Abdala‐Roberts *et al*. 2016). Dioecy, present in ca. 6% of higher plant species and 37% of plant families (Pannell & Barrett 1998), often leads to substantial trait differences between male and female plants (Boecklen *et al*. 1990; Barrett & Hough 2013). These include variations in defence against herbivory (Cornelissen & Stiling 2005), largely shaped by sex‐specific differences in energy allocation to growth and reproduction (Bañuelos *et al*. 2004). Females commonly allocate more resources to reproduction—producing flowers and fruits—which may constrain growth and favour higher investment in defences (Coley *et al*. 1985). In contrast, males typically grow faster and may invest less in defence (Jing & Coley 1990), potentially increasing susceptibility to herbivores (Cornelissen & Stiling 2005). However, recent meta‐analyses and reviews challenge this paradigm (Avila‐Sakar & Romanow 2012; Johnson *et al*. 2015; Juvany & Munné‐Bosch 2015; Sargent & McKeough 2022), reporting species in which males grow more slowly (Bañuelos *et al*. 2004; Massei *et al*. 2006) and invest equally or more in defence than females (Yang *et al*. 2020). These findings reveal the complexity of plant defence strategies and caution against generalizing patterns of sexual dimorphism across taxa.

Part of the variability in sex‐specific herbivory and defence may arise from timing of measurements within the growing season. In many dioecious species, resource allocation between growth and reproduction shifts seasonally (Zhang *et al*. 2016; Tonnabel *et al*. 2017). These temporal dynamics may lead to changing herbivory and defence patterns between the sexes as they adjust allocation priorities. For example, females may focus on vegetative growth early in the season and shift toward reproduction later, altering defence investment and potentially affecting susceptibility to herbivores (Liu *et al*. 2021). Likewise, males may emphasize reproductive output early and reduce defence investment, resulting in greater herbivory later in the season (Agren 1988; Popp & Reinartz 1988; Delph 1990). These temporal shifts complicate herbivory patterns and underscore the need to integrate seasonal perspectives into studies of plant–herbivore interactions. Notably, previous research has relied on single time‐point measurements, and no study has explicitly examined how sex‐specific herbivory and defence vary throughout the growing season—overlooking a critical temporal dimension in ecology of dioecious plants.

Defence traits may help explain observed seasonal and sex‐specific patterns of herbivory. Shifts in traits, such as secondary metabolite concentrations or leaf morphology, likely reflect changing resource allocation priorities across the season, as plants navigate trade‐offs between defence, growth and reproduction (Koricheva & Barton 2012). For instance, elevated concentrations of secondary metabolites early in the season may indicate transient prioritization of defence and photosynthetic efficiency, offering protection during vulnerable development stages (Gaytán *et al*. 2022). Although sex‐related differences in herbivory may not always coincide with significant variation in defence, subtle differences in trait expression could still modulate herbivore pressure across sexes and seasons. These dynamics suggest that defence traits are important components of a temporally variable strategy, and highlight the importance of considering both sex and season when interpreting herbivory patterns. However, to our knowledge, no previous studies have explicitly tested the joint role of sex and seasonal variation in shaping plant defence traits and their consequences for herbivory.

In this study, we investigated the influence of plant sex on herbivory and defence in the dioecious shrub *Pistacia lentiscus* L. (Anacardiaceae) across the growing season in Doñana National Park. We measured insect leaf herbivory and two key leaf traits associated with herbivore resistance – total phenolic content and specific leaf area (SLA) – in 100 *P. lentiscus* plants (53 females, 47 males) at two sites during early and late growing seasonal phases. In this species, phenolics act as chemical defences that deter herbivores or reduce leaf digestibility, while SLA reflects leaf toughness and palatability, with lower values typically indicating greater resistance (Jonasson *et al*. 2003; Landau *et al*. 2010; Navon *et al*. 2020). Our work addresses two main questions: (1) do plant sex effects on herbivory and plant defence vary over the course of the growing season; and (2) are seasonal and sex‐specific patterns of herbivory mediated by variation in defence traits? By incorporating temporal dynamics into the study of herbivory and defence, our findings offer new insights into the role of sexual dimorphism in shaping plant–herbivore interactions in dioecious species.

## MATERIAL AND METHODS

### Natural history

The mastic tree (*P. lentiscus*) is a dioecious evergreen shrub, dominant in Mediterranean lowlands (Martínez‐López *et al*. 2020), and reaching heights of 1–5 m. This species produces new leaves during vegetative growth in early spring, just after flowering, signalling the start of the active growing season. Conversely, the largest loss of old leaves typically occurs in autumn and early winter, as the plant enters a period of dormancy or reduced metabolic activity (Martínez‐Pallé & Aronne 2000). As a keystone species, the mastic tree supports a rich diversity of insect herbivores, especially leaf chewers, skeletonizers and sap‐sucking hemipterans (Davatchi 1958). To defend against herbivores, this plant produces a variety of chemical compounds, including essential oils and phenolics. The oils can have repellent properties, while phenolic compounds may act as antioxidants or inhibit digestion of plant tissues by herbivores (Landau *et al*. 2010; Navon *et al*. 2020). In addition to chemical defences, this plant also possesses physical defences, such as a low SLA (Jonasson *et al*. 2003), which is associated with increased leaf toughness. The leaf traits are usually influenced by environmental factors (Said *et al*. 2011), seasonal changes (Said *et al*. 2011), phenological stages (Carvalho *et al*. 2014), and plant sex (Juvany *et al*. 2014; Yaniv & Dudai 2014).

### Study site

**Fig. 1:**
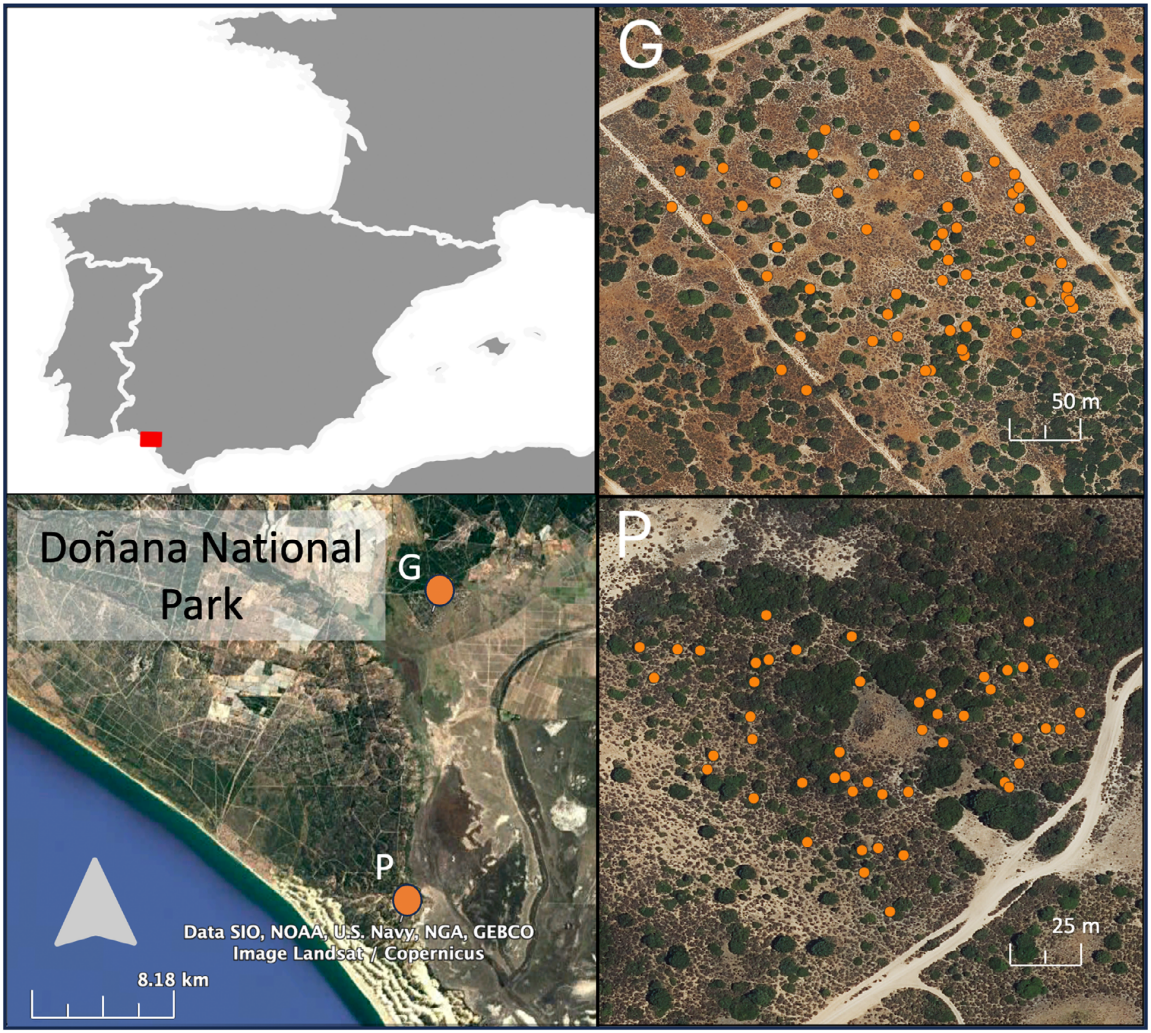
Maps showing location of Doñana National Park and the two study sites, El Puntal (P) and Matasgordas (G), are provided in the left panels. Right panels display spatial distribution of sampled *P. lentiscus* individuals within each site, indicated by orange circles. These maps provide a visual overview of the study area and geographic positioning of the sampled plants.

The field experiment was conducted in Doñana National Park, southern Spain. A total of 100 *P. lentiscus* plants were selected across two Mediterranean scrubland sites: El Puntal (P; 36°57′54.38″N, 6°26′47.15″W) and Matasgordas (G; 37°07′28.88″N, 6°25′48.71″W; Fig. 1). Both sites are Mediterranean sclerophyllous shrubland dominated by *P. lentiscus*. Other species present in the area include *Phillyrea angustifolia*, *Olea europaea* var. *sylvestris*, *Asparagus aphyllus*, *Myrtus communis, Erica arborea, Ulex australis, Halimium halimifolium* and *Cistus salviifolius*. In June (i.e., early growing season, just after flowering), 92 plants (46 females, 46 males) were sampled; while in October (i.e., late growing season, at the peak of fruiting), 100 *P. lentiscus* plants (53 females and 47 males) were sampled. Eight plants were not sampled in June because they lacked sufficient leaves to assess insect herbivory. We georeferenced each individual *P. lentiscus* plant using a handheld GPS device, and recorded its precise location coordinates. Canopy cover area was then measured in QGIS (v. 3.34.5‐Prizren; Quantum GIS Development Team, 2021) by manually delineating each plant's canopy polygon from high‐resolution aerial imagery obtained from Google Earth (Google Inc., 2024), with an approximate spatial resolution of 0.5 m.

### Herbivory measurements

We randomly collected 20 fully expanded leaves of similar age per plant, based on position along the branch, colour, and texture (Moreira *et al*. 2024), to assess herbivory during both the early (June) and late (October) growing season of 2023. The leaves were oven‐dried immediately after collection for a minimum of 48 h at 45°C. For each leaf, we visually estimated insect herbivory as the percentage of leaf area removed by leaf chewers and skeletonizers, using eight defoliation categories: 0%, 0.1%–5%, 5.1%–10%, 10.1%–15%, 15.1%–25%, 25.1%–50%, 50.1%–75%, and >75.1%. To minimize bias, a trained observer (EVC) conducted the assessments without knowledge of the origin of the leaf. Herbivory was then averaged at plant level using the midpoint of each percentage category to calculate a mean value for each plant. Although deer are present in our field sites, we did not observe any vertebrate herbivory damage on the sampled plants.

### Leaf trait estimation

We collected 10 fully expanded, undamaged leaves per plant during the early (June) and late (October) seasons to analyse defence traits (phenolics and SLA), using the same criteria as for herbivory sampling: leaf position, colour and texture. We measured leaf traits on undamaged leaves to minimize confounding effects from induced defences, especially local induction triggered by prior herbivory (Abdala‐Roberts *et al*. 2016; Moreira *et al*. 2024). The leaves were oven‐dried for 48 h at 45°C. For each plant, we finely grounded five oven‐dried leaves to obtain a single sample per plant. We then extracted 20 mg leaf tissue with 70% methanol in an ultrasonic bath for 15 min, followed by centrifugation and dilution of the methanolic extract (Moreira *et al*. 2014). We colorimetrically determined total phenolic content using the Folin–Ciocalteu method in a Biorad 650 microplate reader (Bio‐Rad Laboratories, PA, USA) at 740 nm, with tannic acid as standard (Moreira *et al*. 2014). The remaining five leaves were scanned and weighed to calculate specific leaf area (SLA, cm^2^ mg^−1^). Leaf area was determined using ImageJ v. 2.14.0/1.54f (ImageJ2, 2023), and SLA was averaged at plant level.

### Statistical analysis

We built Linear Mixed‐effect Models (LMM) to analyse the effects of plant sex (two levels: male vs. female), season (two levels: early vs. late), and their interaction, as well as site (El Puntal vs. Matasgordas) (all fixed factors) on insect herbivory and leaf defences (phenolics and SLA). We also included plant area as a covariate to assess whether the size of the plant influenced herbivory levels and leaf traits. To account for repeated measurements from the same plants across different seasons, plant ID was included as a random factor in the models.

To evaluate whether assumptions of the LMMs were met, we conducted diagnostic checks of residuals. Specifically, we examined residuals versus fitted values to assess homoscedasticity, and used Q–Q plots and kernel density plots of residuals to evaluate normality. These visual inspections indicated that the assumptions of normality and homoscedasticity were reasonably satisfied for both phenolics and herbivory data. To further validate these results, we employed functions from the *performance* package (e.g., check_model() and check_distribution()). Model fit was assessed by calculating marginal and conditional *R* ^2^ values using the r2() function from the same package. These metrics represent variance explained by the fixed effects alone (marginal *R* ^2^) and by both fixed and random effects combined (conditional *R* ^2^). To enhance transparency and allow readers to assess model robustness, diagnostic plots for the main models are provided in the Figs. S1 and S2.

Additionally, we conducted a permutation‐based ANOVA (aovperm) to assess the effects of the same studied factors on SLA. The aovperm method was chosen because of the non‐normal distribution of the data and was used with 5000 permutations and the Freedman‐Lane method to account for nuisance variables. In contrast, total phenolic content data had approximately normal distribution, and diagnostic checks indicated that model residuals met the assumptions of normality and homoscedasticity. Therefore, we retained the LMM approach for this variable as it provided a parsimonious and interpretable analytical framework. To ensure consistency and assess the robustness of our results, we additionally conducted a sensitivity analysis using permutation‐based ANOVA (aovperm) for phenolics. This alternative analysis yielded qualitatively similar results (not shown), further supporting the validity and reliability of the LMM‐based findings.

Finally, to evaluate whether the measured leaf traits mediated the effects of plant sex and season on herbivory, we re‐ran the herbivory model described above, this time including total phenolics and SLA – measured separately for the early and late seasons – as covariates. We expected that if physical traits or chemical defences mediate effects of plant sex and season on leaf herbivory, then significant effects of any of these factors (or their interaction) should be non‐significant once such traits are accounted for in the model.

We focused on how leaf traits influenced herbivory, rather than the reverse, because our measurements were based on undamaged leaves. This approach allowed us to assess constitutive defences, minimizing potential bias from locally induced responses triggered by herbivore damage, and better isolating intrinsic variation in plant defence strategies (Abdala‐Roberts, Moreira, *et al*. 2016; Moreira *et al*. 2024). Pairwise differences between levels of the interaction (Plant sex × Season) were tested using post‐hoc pairwise comparisons of estimated marginal means, adjusted for multiple comparisons with the Tukey method (performed with the emmeans package in R).

Prior to modelling, we standardized and centered all continuous predictors to facilitate comparisons of their effect sizes. We also checked for multicollinearity by calculating the variance inflation factor (VIF) for each explanatory variable, ensuring that none had strong correlations (all VIFs <5, the standard threshold for detecting multicollinearity issues; Miles 2022). All analyses were run in the R Core Team (2024) with packages lme4 (Bates *et al*. 2014), performance (Lüdecke *et al*. 2021), emmeans (Lenth 2024), car (Fox & Weisberg 2018), permute (Simpson 2022) and permuco (Frossard & Renaud 2021).

## RESULTS

**Fig. 2:**
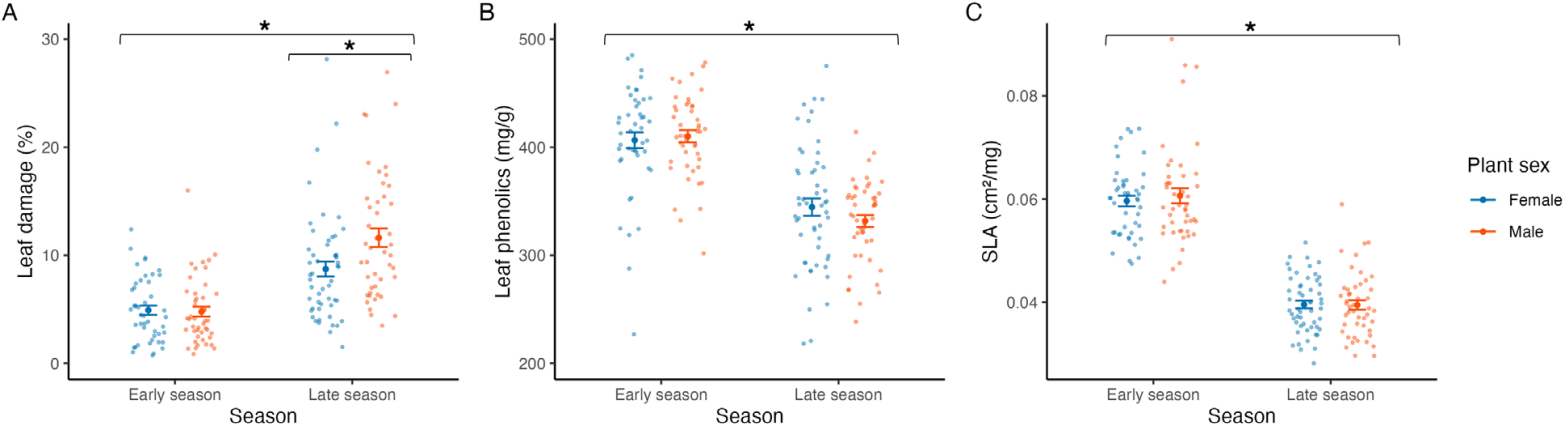
Variations in insect herbivory (A), leaf phenolics (B) and specific leaf area (SLA) (C) between the early and the late season, and between female and male plants. Small dots represent raw data, while large dots and error bars represent mean values of the raw data ± SE. Asterisks indicate significant differences between seasons (LMM and aovperm) and between sexes (LMM followed by post hoc pairwise comparisons).

Insect herbivory caused an average (± SE) of 8.05 ± 0.48% damage to leaf area across sampled plants (*n* = 92 in the early season and *n* = 100 in the late season). We found that plant sex and season significantly affected leaf herbivory. Herbivory was significantly higher in males (mean ± SE: 8.79 ± 0.74%) than in females (7.35 ± 0.62%), and in the late season (11.00 ± 0.77%) compared to the early season (4.84 ± 0.31%). We also found a significant plant sex × season interaction (Table 1), where insect herbivory was significantly higher on males than on females in the late season (mean difference = 0.269 ± 0.102 SE, *P*‐value = 0.045 from the post‐hoc pairwise comparisons; Fig. 2A), while there were no significant differences in the early season (mean difference = −0.014 ± 0.105, *P*‐value = 0.999 from post‐hoc pairwise comparisons; Fig. 2A).

**Table 1:** Summary of linear mixed models testing the effect of plant sex (two levels: male vs. female), season (two levels: early vs. late), their interaction, plant area, and site (two levels: El Puntal vs. Matasgordas) on insect herbivory and leaf phenolics.

predictor	*F‐*value (df)	*P‐*value	estimate (SE)	*R* ^2^_m_ (*R* ^2^_c_)
Insect herbivory				
Plant sex	2.642 (1)	**<0.001**	−0.268 (0.102)	0.369 (0.621)
Season	−6.989 (1)	**<0.001**	−0.559 (0.080)	
Plant sex × season	2.462 (1)	**0.0156**	0.282 (0.115)	
Plant area	3.025 (1)	**<0.001**	0.008 (0.003)	
Site	0.144 (1)	0.886	0.012 (0.085)	
Leaf phenolics				
Plant sex	−1.777 (1)	0.077	−16.851 (9.482)	0.381 (0.539)
Season	7.125 (1)	**<0.001**	59.015 (8.283)	
Plant sex × season	1.704 (1)	0.092	20.215 (11.864)	
Plant area	−0.624 (1)	0.534	−2.370 (3.799)	
Site	3.271 (1)	**<0.010**	24.797 (7.582)	

*P*‐values are in bold when they are significant. Marginal (*R* ^2^_m_) and conditional (*R* ^2^_c_) *R* ^2^ are reported.

The concentration of leaf phenolics was, on average, 372.79 ± 4.29 mg g^−1^. Season, but not plant sex or the plant sex × season interaction, significantly affected leaf phenolics (Table 1). In particular, the concentration of leaf phenolics was higher in the early season (408.32 ± 4.64 mg g^−11^) than in the late season (304.11 ± 5.23 mg g^−1^) (Fig. 2B). The SLA was, on average, 4.954 ± 0.089 mm^2^ mg^−1^. Similarly, season, but not plant sex or the plant sex × season interaction, significantly affected SLA (Table 2). Specifically, SLA was significantly higher in the early season (6.01 ± 0.09 mm^2^ mg^−1^) than in the late season (3.95 ± 0.06 mm^2^ mg^−1^; Fig. 2C).

**Table 2:** Summary of aovperm, testing the effects of plant sex, season, their interaction, plant area and site on SLA.

predictors	SS	*F‐*value (df)	*P‐*value
Plant sex	0.04	0.074 (1)	0.790
Season	203.4	380 (1)	**<0.001**
Plant sex × season	0.141	0.263 (1)	0.605
Plant area	0.001	0.002 (1)	0.967
Site	0.028	0.053 (1)	0.825

*P*‐values are in bold when they are significant. Sum of Squares (SS) is the variability explained by each factor.

When leaf phenolic concentration and SLA were included as covariates in the model for herbivory, the effects of plant sex, season and their interaction remained significant (Table 3), suggesting that these leaf traits did not account for the observed plant sex and season effects on herbivory.

**Table 3:** Summary of linear mixed models testing the effects of plant sex, season, their interaction, leaf phenolics, plant area, SLA and site on insect herbivory.

predictor	*F‐*value (df)	*P‐*value	estimate (SE)	*R* ^2^_m_ (*R* ^2^_c_)
Plant sex	2.432 (1)	**0.016**	0.250 (0.103)	0.371 (0.630)
Season	−3.769 (1)	**<0.001**	−0.499 (0.132)	
Plant sex × season	−2.256 (1)	**0.026**	−0.259 (0.115)	
Plant area	2.947 (1)	**<0.010**	0.127 (0.043)	
Site	0.446 (1)	0.656	0.039 (0.088)	
Leaf phenolics	−1.426 (1)	0.156	−0.064 (0.045)	
SLA	0.036 (1)	0.971	0.002 (0.059)	

*P*‐values are in bold when they are significant. Marginal (*R* ^2^_m_) and conditional (*R* ^2^_c_) *R* ^2^ are reported.

## DISCUSSION

This study highlights the importance of temporal dynamics in the relationship between plant sex and herbivory in *P. lentiscus*. While most previous research focused on static comparisons between male and female plants, our study is, to our knowledge, the first to explicitly investigate how these interactions shift across the growing season. By examining both early and late season patterns, we show that herbivory levels – but not defence traits – are shaped by a dynamic interplay between plant sex and seasonality. These findings highlight the need to consider seasonal context to fully understand sex‐specific ecological strategies and plant–herbivore interactions in dioecious species.

We found that herbivory was higher in male plants than in female plants. These findings align with previous studies suggesting that male plants in dioecious species may be more vulnerable to herbivory because of a trade‐off between growth and defence (Jing & Coley 1990; Cornelissen & Stiling 2005). Males often prioritize vegetative growth, potentially limiting investment in chemical or structural defences, particularly during periods of high herbivore pressure (Yang *et al*. 2020). However, contrary to our expectations, we found no direct effects of plant sex on phenolic concentrations or SLA – two traits commonly associated with herbivore resistance. Although both phenolics and SLA are widely recognized as important determinants of herbivore damage (Agrawal 2007; Mithöfer & Boland 2012), the literature reports mixed results regarding their effectiveness and sex‐based variation (Cornelissen & Stiling 2005). Our findings are consistent with studies that also report no sexual dimorphism in defence traits: Stark & Martz (2018) found no sex‐based differences in phenolic or terpenoid concentrations in *Juniperus communis*, and Nell *et al*. (2018) reported no differences in SLA, C:N ratio, water content, toughness or terpene levels in *Baccharis salicifolia*. Similarly, Hjältén *et al*. (1993) found no sex‐based differences in leaf N content in *P. lentiscus*. These results suggest that herbivory differences between sexes may not be explained by commonly measured traits alone, pointing to the involvement of other, possibly unmeasured, mechanisms, such as induced responses, volatile organic compounds, or herbivore behaviour. Future studies should investigate these additional pathways to better understand the complexity of plant–herbivore interactions in dioecious systems.

Herbivory was significantly higher in the late season compared to the early season, reflecting an increase in herbivore activity as the growing season progresses. This is likely related to the rise in insect populations, especially toward late summer and early autumn, as many herbivores complete their reproductive cycles and have higher numbers of individuals foraging on plants (Richards & Coley 2007; Wang *et al*. 2023). Moreover, the cumulative effect of herbivory over time likely contributes to the increased damage seen in the late season, as plants face continuous herbivore pressure throughout the growing period. For instance, Richards & Coley (2007) found a significant increase in herbivore activity and damage during the late season in tropical dry forests. Similarly, Wang *et al*. (2023) found a significant increase in herbivory in late season, especially in deciduous broadleaved seedlings. In contrast, phenolic concentrations were higher in the early season, supporting the idea that plants prioritize chemical defences when herbivore pressure is lower (Carvalho *et al*. 2014). Phenolic compounds, which serve as a deterrent to herbivores, are typically more concentrated during rapid growth phases when resource availability is high (Said *et al*. 2011). Similarly, SLA was higher in the early season than in the late season, reflecting the production of thinner, faster‐growing leaves that shift toward tougher, thicker leaves in the late season (Reich 1991; Zhang *et al*. 2016). This change in leaf structure makes plants more resistant to herbivory as the season progresses. These seasonal shifts in leaf traits and defences illustrate the dynamic nature of plant strategies, where plants adjust resource allocation to balance growth and protection in response to seasonal changes and herbivore pressure (Coley *et al*. 1985; Tonnabel *et al*. 2017).

The interaction between plant sex and season was a key finding in this study, significantly influencing herbivory patterns. Male plants experienced higher herbivory than female plants in the late season, but there were no differences was in the early season. This dynamic interaction likely reflects shifting resource allocation priorities as dioecious plants transition from vegetative to reproductive stages. Males may prioritize growth early in the season, investing less in defence, while females, which allocate more resources to reproduction, may increase investment in defence traits later in the season (Tonnabel *et al*. 2017). These temporal shifts underscore the importance of considering both plant sex and seasonal changes to understand herbivory patterns in dioecious species. Notably, despite this observed interaction, the plant sex × season effect did not influence the defence traits measured in this study, namely phenolic concentration and SLA. This highlights the need to investigate additional forms of defence beyond those measured in the present study, including structural traits (e.g. trichomes), inducible responses and tolerance strategies, such as regrowth capacity or nutrient mobilization. Furthermore, examining seasonal variation in herbivore identity and feeding behaviour could help determine whether changes in herbivore communities drive the observed patterns (Avila‐Sakar & Romanow 2012; Juvany *et al*. 2014). Future studies addressing these aspects would provide a more comprehensive understanding of the temporal dynamics underlying plant–herbivore interactions, and offer valuable insights into the ecological consequences of sexual dimorphism in dioecious species.

This study highlights the temporally dynamic nature of plant–herbivore interactions in dioecious species, emphasizing the critical role of seasonal variation. These findings underscore the complexity of herbivory and defence strategies in *P. lentiscus*, showing that these processes fluctuate throughout the growing season, rather than remaining static. Further research is needed to explore additional, unmeasured traits, such as trichome density and volatile organic compounds, which may influence herbivore behaviour. Additionally, incorporating abiotic factors, such as climate variability or soil characteristics, will provide further insights into how these factors interact with plant traits to shape herbivore dynamics. A more integrated approach will improve our understanding of the complex, shifting relationships between plants and herbivores across different ecological contexts.

## AUTHOR CONTRIBUTIONS

EVC, GC and PJ selected the study sites. EVC and GC selected the trees and collected the leaves in the field. EVC measured canopy cover, insect herbivory and SLA. GC, CR, BLN and XM measured total phenolics. EVC analysed the data. EVC and XM led the writing, and all authors contributed critically to the revisions. All authors wrote the final version of the manuscript.

## Supporting information


**Fig. S1.** Diagnostic plots for residuals of the Linear Mixed‐effects Model applied to leaf damage data. Plots include (top left) residuals versus fitted values to assess homoscedasticity, (top right) Q–Q plot of residuals to check normality, (bottom left) density plot of residuals, and (bottom right) Q–Q plot of fitted values. Overall, residuals met assumptions of normality and homogeneity of variance.
**Fig. S2.** Diagnostic plots for residuals of the Linear Mixed‐effects Model applied to total phenolics concentration data. Plots include (top left) residuals versus fitted values to assess homoscedasticity, (top right) Q–Q plot of residuals to check normality, (bottom left) density plot of residuals, and (bottom right) Q–Q plot of fitted values. Overall, residuals met assumptions of normality and homogeneity of variance.
