# Supplementary material for: Season over plant sex: drivers of leaf damage and plant defence in a dioecious Mediterranean shrub
Source: Plant Biol (Stuttg). 2025 Sep 15;28(1):314–20. doi: 10.1111/plb.70115 (PMC12710833; doi:10.1111/plb.70115)
Supplement: Supplementary file 1 — Fig. S1. Diagnostic plots for residuals of the Linear Mixed‐effects Model applied to leaf damage data. Plots include (top left) residuals versus fitted values to assess homoscedasticity, (top right) Q–Q plot of residuals to check normality, (bottom left) density plot of residuals, and (bottom right) Q–Q plot of fitted values. Overall, residuals met assumptions of normality and homogeneity of variance. Fig. S2. Diagnostic plots for residuals of the Linear Mixed‐effects Model applied to total phenolics concentration data. Plots include (top left) residuals versus fitted values to assess homoscedasticity, (top right) Q–Q plot of residuals to check normality, (bottom left) density plot of residuals, and (bottom right) Q–Q plot of fitted values. Overall, residuals met assumptions of normality and homogeneity of variance. [file PLB-28-314-s001.docx]

**Supplementary material**


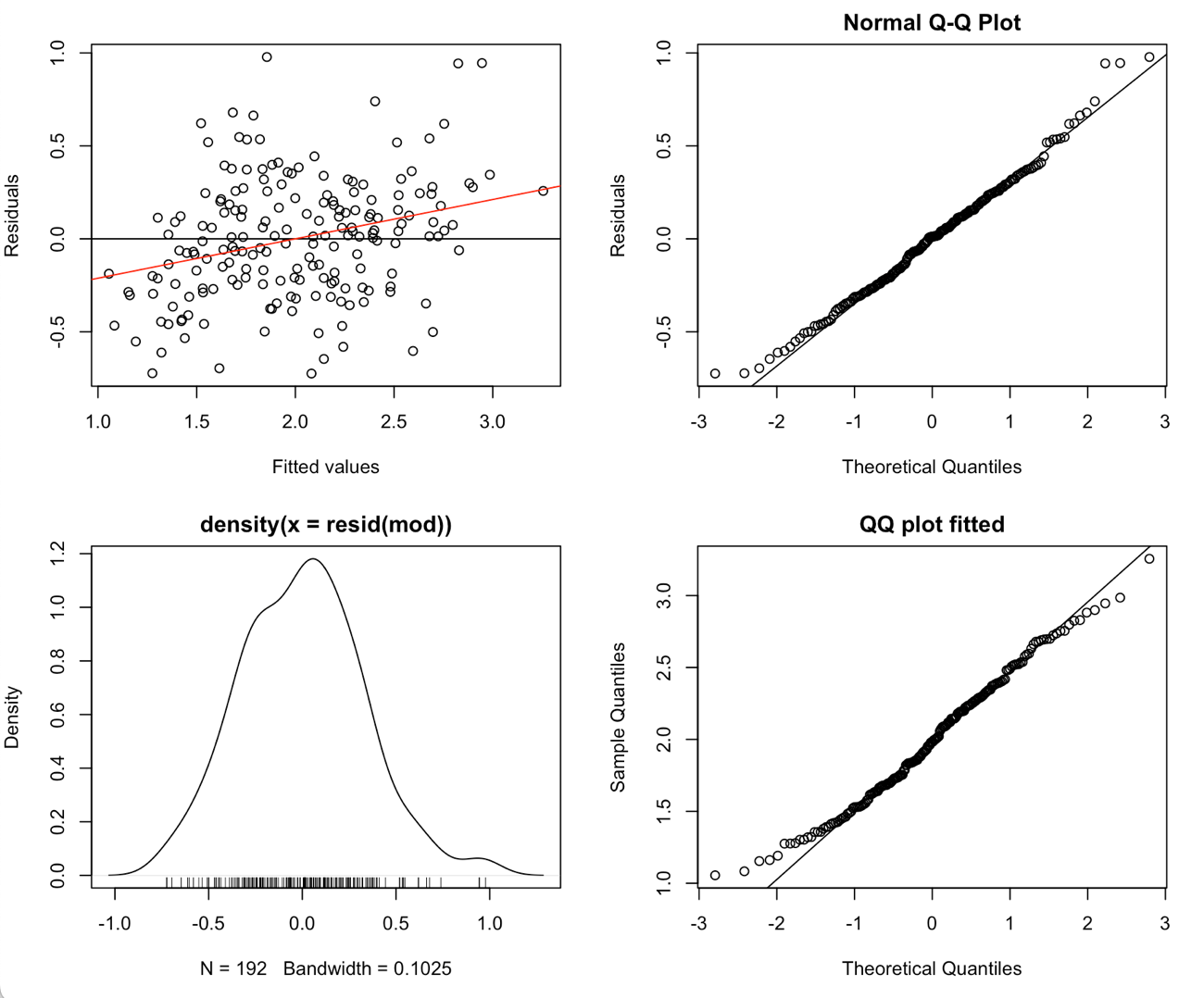
**Figure S1**. Diagnostic plots for residuals of the Linear Mixed-effects Model applied to leaf damage data. Plots include (top left) residuals versus fitted values to assess homoscedasticity, (top right) Q–Q plot of residuals to check normality, (bottom left) density plot of residuals, and (bottom right) Q–Q plot of fitted values. Overall, residuals met assumptions of normality and homogeneity of variance.


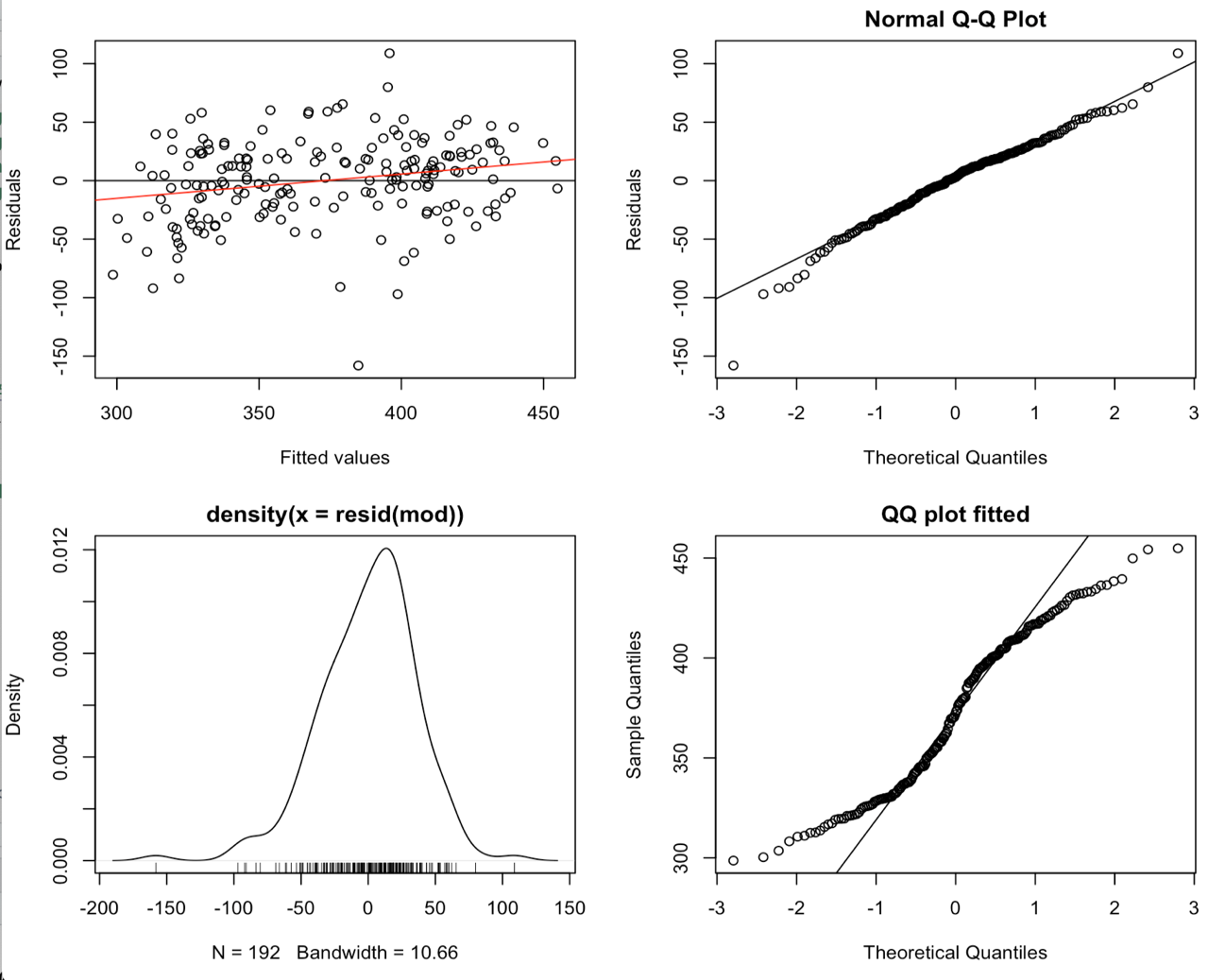


**Figure S2**. Diagnostic plots for residuals of the Linear Mixed-effects Model applied to total phenolics concentration data. Plots include (top left) residuals versus fitted values to assess homoscedasticity, (top right) Q–Q plot of residuals to check normality, (bottom left) density plot of residuals, and (bottom right) Q–Q plot of fitted values. Overall, residuals met assumptions of normality and homogeneity of variance.
